# A theory of power laws in human reaction times: insights from an information-processing approach

**DOI:** 10.3389/fnhum.2014.00621

**Published:** 2014-08-12

**Authors:** José M. Medina, José A. Díaz, Kenneth H. Norwich

**Affiliations:** ^1^Departamento de Óptica, Facultad de Ciencias, Universidad de GranadaGranada, Spain; ^2^Department of Physics, Institute of Biomaterials and Biomedical Engineering, University of TorontoToronto, ON, Canada

**Keywords:** human reaction time, intrinsic variability, power laws, information transfer, Piéron's law

Human reaction time (RT) can be defined as the time elapsed from stimulus presentation until a reaction/response occurs (e.g., manual, verbal, saccadic, etc.). RT has been a fundamental measure of the sensory-motor latency at suprathreshold conditions for more than a century and is one of the hallmarks of human performance in everyday tasks (Luce, 1986; Meyer et al., 1988). Some examples are the measurement of RTs in sports science, driving safety or in aging. Under repeated experimental conditions the RT is not a constant value but fluctuates irregularly over time. Stochastic fluctuations of RTs are considered a benchmark for modeling neural latency mechanisms at a macroscopic scale (Luce, 1986; Smith and Ratcliff, 2004). Power-law behavior has been reported in at least three major types of experiments. (1) RT distributions exhibit extreme values. The probability density function (pdf) is often heavy-tailed and can lead to an asymptotic power-law distribution in the right tail (Holden et al., 2009; Moscoso del Prado Martín, 2009; Sigman et al., 2010). (2) RT variability (e.g., variance) is not bounded and usually shows a power relation with the mean, with an exponent β close to unity (Luce, 1986; Wagenmakers and Brown, 2007; Holden et al., 2009; Medina and Díaz, 2011, 2012). This relationship is a manifestation of Taylor's law (also called “fluctuation scaling”) (Taylor, 1961; Eisler et al., 2008), although departures from power law have been reported (Eisler et al., 2008; Schmiedek et al., 2009). And (3), the mean RTs decay as the stimulus strength increases (Cattell, 1886), an issue that is well-described by a truncated power function written in the form of Piéron's law (Piéron, 1914, 1920; Luce, 1986):

(1) $$t_{n+1}=t_{n}+\frac{d}{S^{p}}$$

*t*_*n* + 1_ indicates the mean RT, *S* is the stimulus strength (e.g., loudness intensity, odor concentration, etc.), *t_n_* represents the asymptotic component of the mean RT reached at very high stimulus strength and *d* and *p* are two parameters (Luce, 1986). The sub-index *n* denotes the time step or order and it indicates a causal process: *t*_*n* + 1_ grows from the previous stage *t_n_* by an additive factor that depends on the stimulus strength *S* (Medina, 2009). The previous stage *t_n_* contains those processes at the threshold at an earlier time and *t*_*n* + 1_ in Equation (1) describes those processes at suprathreshold conditions at a later time (Norwich et al., 1989; Medina, 2009). The origin of power-law behavior in RTs has been a long-standing issue. Considerable effort has been dedicated in modeling each power relation separately. While it might be plausible that power laws in RTs could share a limited number of mechanisms, a successful theory remains unresolved. The ubiquity of power laws in many biological and physical systems has revealed the existence of multiple generative mechanisms (Mitzenmacher, 2004; Newman, 2005; Sornette, 2007; Frank, 2009). Research on a unifying framework that links power laws in RTs is an important issue for better understanding the emergent complex behavior of neural activity in simple decisions and in dysfunctional states.

We propose that type (3) power laws govern the threshold for RT; and it follows consequently that power laws govern suprathreshold fluctuations in RT. Piéron's law is valid for each sensory modality (Chocholle, 1940; Banks, 1973; Luce, 1986; Overbosch et al., 1989; Pins and Bonnet, 1996; Bonnet et al., 1999), and in both simple and choice reaction times (Schweickert et al., 1988; Pins and Bonnet, 1996). Instead of diffusion models (Luce, 1986; Smith and Ratcliff, 2004), we use elements from information theory and statistical physics as the principal conceptual tools. We also discuss random multiplicative processes as an important approach to Piéron's law and power laws in RTs.

In our information-theoretic formalism, the information entropy function *H* always expresses a measure of uncertainty within a sensory neural network. High information entropy values indicates high uncertainty and vice versa. Information is related to the drop of uncertainty (measured, e.g., in bits). It is postulated that sensory perception is not an instantaneous act but it always takes time (Norwich, 1993). Initially, for a given external input signal, the sensory system encodes the stimulus efficiently and then, it adapts and transfers information over time. Therefore, the *H*-function depends explicitly on the time to represent a continuous process of sensory adaptation (Norwich, 1993). The human RT can be re-defined as the time needed to accumulate ΔH bits of information after efficient encoding (Norwich et al., 1989; Norwich, 1993):

(2) $$\Delta H=H\left(\frac{1}{t_{0}}\right)-H\left(\frac{1}{t_{n+1}}\right)>0$$

Figure 1A represents the entropy function *H* in Equation (2). At least two stages can be differentiated. The *H*-function evolves from a previous state of maximum uncertainty reached at the encoding time *t*_0_, *H* (1/*t*_0_), to a final adapting stage with a lower uncertainty *H* (1/*t*_*n* + 1_) where a reaction occurs, (*t*_*n* + 1_ >*t*_0_). Maximum production of entropy and then, a reduction of uncertainty in ΔH as a function of time are concepts introduced from statistical physics, the latter as expressed by Boltzmann (Norwich, 1993). Based on an analytical model of the *H*-function (Norwich, 1993), the gain of information ΔH is connected with the formation of an internal threshold in Equation (1) (Norwich et al., 1989; Medina, 2009):

**Figure 1:**
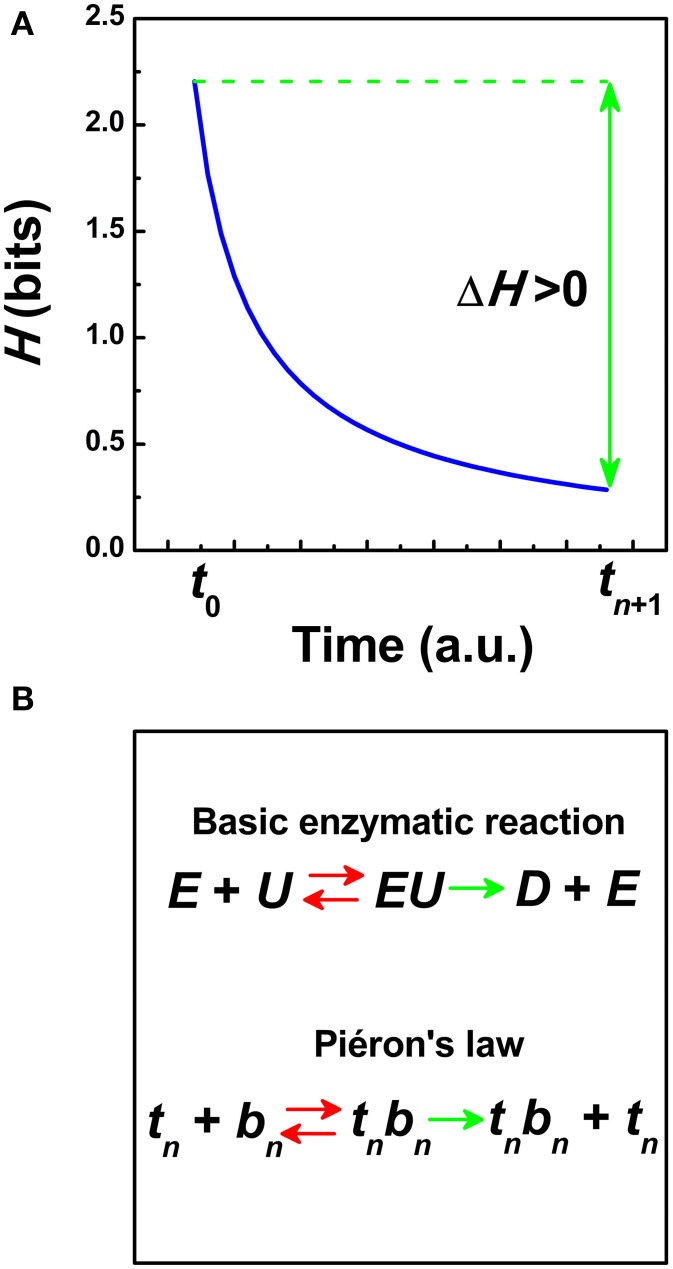
**(A)** Schematic representation of the information entropy function *H* (1/*t*) (in bits) as a function of the time *t* (Norwich, 1993). The transfer of information ΔH is defined in Equation (2) from the encoding time *t*_0_ until a reaction occurs at *t*_*n* + 1_. (a.u.) = arbitrary units. **(B)** Schematic representation of a model of hyperbolic growth in reaction times based on Piéron's law and analogous to Michaelis-Menten kinetics in biochemistry (i.e., the Hill equation) (Pins and Bonnet, 1996). In Michaelis-Menten kinetics, an enzyme *E* is bounded to a substrate *U* to form a complex *EU* that is converted into a product *D* and the enzyme *E*. In Piéron's law, those neurons tuned at the time *t_n_* are bounded to those neurons that perform the formation of an internal threshold *S*_0_ in *b_n_* = (*S*_0_/*S*)^*p*^ to form the term *t*_*n*_ b_n_ that is converted into the product *t*_*n*_ b_n_ plus the time *t_n_*. Red double arrows indicate that the “reaction” is reversible whereas green single arrows indicate that the “reaction” goes only in one way.

(3) $$d=t_{n}S_{0}^{p}$$

Piéron's law can be written as follows:

(4) $$t_{n+1}=\left(b_{n}+1\right)t_{n},$$

where *b_n_* = (*S*_0_/*S*)^*p*^. The parameter *S*_0_ represents an estimation of the internal threshold that controls the RT: an external incoming signal *S* exceeding *S*_0_ leads to a RT response (Norwich et al., 1989). Furthermore, *S*_0_ varies based on several factors and provides the sensitivity (1/*S*_0_) of the sensory system (e.g., in vision the human contrast sensitivity function) (Felipe et al., 1993; Murray and Plainis, 2003). The model of Piéron's law in Equation (4) sets a number of important properties. First property, Equation (4) indicates the existence of multiplicative interactions in a cascade between different time scales: the mean RT is expressed in terms of the asymptotic time, *t_n_*, and Piéron's law is written in multiples of threshold *S*_0_. That is, we work with dimensionless ratios of *S*_0_/*S* (Norwich, 1993). Different interpretations of the exponent *p* have been reported. *S*^*p*^_0_ could be interpreted as the transfer or transducer function between neurons (Copelli et al., 2002; Billock and Tsou, 2011) at the threshold. The exponent *p* usually takes non-integer values and could indicate a signature of self-organized criticality in a phase transition (Kinouchi and Copelli, 2006). Here the concept of phase transition does not deal with the classical view of different states of matter in thermodynamics (e.g., liquid vs. gas), but with different states of connectivity between neurons as modeled by branching processes (Kinouchi and Copelli, 2006). Alternatively, power functions *S*^*p*^_0_ can be derived from Mackay transforms (Mackay, 1963) and the exponent *p* could represent oscillatory synchronization states between neurons (Billock and Tsou, 2005, 2011). The model of Piéron's law in Equation (4) is a useful alternative approach and optimal information transfer is related with the entropy function *H* (Norwich, 1993). Low values of *p* will promote a minimum in ΔH after efficient encoding, i.e., an *Infomin* principle at the macroscopic scale (Medina, 2011, 2012).

Second property, the threshold barrier *S*_0_ is not a fixed static value but unstable and fluctuates over time due to the presence of endogenous or internal noise (Faisal et al., 2008). Consequently, RTs are influenced and modified by neural noise. Therefore, Equation (4) is not deterministic and is included in a general class of discrete-time stochastic equations that has been used in many applications such as in epidemics, finance, etc. (Levy and Solomon, 1996; Sornette and Cont, 1997; Takayasu et al., 1997; Newman, 2005; Sornette, 2006). The term *b_n_* is a random and positive multiplicative factor that depends on the temporal fluctuations of *S*_0_ and thus, on ΔH. It has been demonstrated that the model of Piéron's law in Equation (4) produce type (1) power laws. RT pdfs obey a transition from a log-normal distribution into a power law in the right tail (Medina, 2012). If RTs are longer than the asymptotic term, *t_n_*, the RT pdf is distributed as a power law with an exponent *γ* that depends on the exponent *p* of Piéron's law (Medina, 2012):γ = 1 + (*c/p*), *c* being a constant. Two different regimes are observed: for those values *p* > 0.6 the central moments diverge and if *p* ≤ 0.6 they are finite (Medina, 2012). Therefore, long RTs compared to the asymptotic term *t_n_* are considered intermittent events over time. Their distribution is characterized by power law pdfs that might have finite or infinite variance. A cautionary note should be mentioned here. The magnitude of *p* could also depend on the metric of the stimulus strength *S* selected and values different from the boundary *p* ≅ 0.6 might be possible. For instance, this is important when testing power law RT pdfs in color vision because an appropriate color contrast metric has not been established (Medina and Diaz, 2010).

Third property, the reciprocal of Piéron's law is invariant under rescaling (Chater and Brown, 1999; Medina, 2009). Taking the reciprocal of the mean RT, *R* = 1/*t*_*n* + 1_. and the reciprocal of the irreducible asymptotic term, *R*_max_ = 1/*t_n_* in Equation (4), then, *R* = *R*_max_ [1 + (*S*_0_/*S*)^*p*^]. Therefore, the reciprocal of the Equation (4) defines an affine transformation over multiple time scales that can be mapped into the Naka-Rushton equation at the cellular level (Naka and Rushton, 1966) and the Michaelis-Menten equation in enzyme reactions at the sub-cellular level (Michaelis and Menten, 1913; Pins and Bonnet, 1996). This suggests that some general properties of RT patterns governed by Piéron's law could be mirrored in part into the dynamics of the Naka-Rushton equation and/or the Michealis kinetics (Medina, 2009, 2012). The Naka-Rushton equation represents a canonical form of non-linear gain control in neural responses before saturation (Albrecht and Hamilton, 1982; Billock and Tsou, 2011; Carandini and Heeger, 2012). Threshold normalization in the Naka-Rushton equation is often modeled as a pool of many neurons tuned to different stimulus properties (Heeger, 1992; Carandini and Heeger, 2012). In the Michaelis-Menten equation, the normalization factor is the Michaelis constant and indicates the substrate concentration at a reference value. The Michaelis constant is related with the substrate's affinity for the enzyme and depends on many factors (Murray, 2002). Figure 1B represents a schematic model of RT growth based on Piéron's law and an analogy with enzyme kinetics.

The exponent *p* of Piéron's law could be related to the scaling exponent β of the variance-mean relationship in type (2) power law. A power law relationship between variance and mean of the stimulus population has been proposed in the *H*-function (Norwich, 1993) and this relationship could be compatible with the RT variance-mean relationship in the regime around *p* > 0.6 (Medina, 2011, 2012). Alternative approaches have explored *α*-stable processes to relate type (1) power laws and long-range correlations (Ihlen, 2013). Tweedie exponential dispersion models are also able to describe type (2) power laws in many biological and physical processes (Eisler et al., 2008; Kendal and Jørgensen, 2011; Moshitch and Nelken, 2014). However, a connection between Piéron's law and *α*-stable and Tweedie models remains unknown.

In summary, maximum entropy *H* and then, adaptation over time in Equation (2) leads to a type (3) power law, Piéron's law Equation (1). The *H*-function also explains many empirical relations of sensory perception (Norwich, 1993). An important message of the entropy function *H* is that the term *d* in Piéron's law depends explicitly on a sensory threshold *S*_0_ by the power law Equation (3). There is also experimental evidence that RTs and threshold-based sensitivities are mediated by common sensory processes (Felipe et al., 1993; Murray and Plainis, 2003). Therefore, temporal fluctuations at the sensory threshold *S*_0_ affect RT fluctuations at suprathreshold conditions and this can be described by means of a simple random multiplicative process in Equation (4). The same multiplicative process produces non-Gaussian RT distributions and type (1) power law RT pdfs. The model of Piéron's law in Equation (4) also generates fractal-like behavior that extends to smaller time scales. The reciprocal of Equation (4) provides a direct link with neural gain control in single neurons as exemplified by the Naka-Ruston equation and a possible analogy with enzyme kinetics within neurons as exemplified by the Michaelis-Menten kinetics.

## Conflict of interest statement

The authors declare that the research was conducted in the absence of any commercial or financial relationships that could be construed as a potential conflict of interest.
